# Vitamin A deficiency in India and seasonality of vitamin A-rich food consumption

**DOI:** 10.1017/S0007114525103681

**Published:** 2025-06-28

**Authors:** Rupinder Sahota, Fanny Sandalinas, Christopher Chagumaira, Robert Johnston, Jaswant S. Khokhar, R. Murray Lark, Arindam Das, Edward J. M. Joy, E. Louise Ander

**Affiliations:** 1 Department of Population Health, London School of Hygiene & Tropical Medicine, London, UK; 2 School of Biosciences, University of Nottingham, Sutton Bonington, UK; 3 UNICEF, New Delhi, India; 4 Centre for Environmental Geochemistry, British Geological Survey, Keyworth, UK; 5 Institute of Health Management Research, IIHMR University, Jaipur, India

**Keywords:** Vitamin A deficiency, Seasonality, Serum retinol, Survey design, Vitamin A-rich foods

## Abstract

Vitamin A deficiency (VAD) poses significant health risks and is prevalent in children and adolescents in India. This study aimed to determine the effect of seasonal variation and availability of vitamin A-rich (VA-rich) foods on serum retinol in adolescents. Data on serum retinol levels from adolescents (*n* 2297, mean age 14 years) from the Comprehensive National Nutrition Survey (2016–2018) in India were analysed, with VAD defined as serum retinol < 0·7 µmol/L. Five states were selected based on a comparable under-five mortality rate and the seasonal spread of the data collection period. Dietary data from adolescents and children ≤ 4 years old were used to assess VA-rich food consumption. A linear mixed model framework was employed to analyse the relationship between serum retinol, month of the year and VA-rich food consumption, with *a priori* ranking to control for multiple hypothesis testing. Consumption of VA-rich foods, particularly fruits and vegetables/roots and tubers, showed seasonal patterns, with higher consumption during summer and monsoon months. Significant associations were found between serum retinol concentrations and age, month of sampling, consumption of VA-rich foods and fish. VAD prevalence was lowest in August, coinciding with higher consumption of VA-rich fruits and foods. Findings highlight the importance of considering seasonality in assessing VAD prevalence and careful interpretation of survey findings. Intentional design, analysis and reporting of surveys to capture seasonal variation is crucial for accurate assessment and interpretation of VAD prevalence, including during monitoring and evaluation of programmes, and to ensure that public health strategies are appropriately informed.

Vitamin A (VA) is essential for growth, development, immunity and visual functions^(1)^. Individuals with VA deficiency (VAD) are at increased risk of impaired immunity, preventable blindness, anaemia and increased morbidity and mortality from a range of infectious diseases^(2)^. Nutrient-poor diets and recurrent infections may lead to depletion of VA stores in the liver and peripheral tissues, leading to VAD^(3)^. VAD is common in countries where there is a high level of poverty, high incidence of infectious diseases, limited infrastructure and food insecurity^(2)^ and is prevalent in southern Asia^(4)^. Approximately 85 % of VAD children in southern Asia live in India (1·87 million children)^(5,6)^. The primary indicator of VA status in large-scale population surveys is serum retinol concentration^(2)^, and VAD is typically defined by serum retinol level below 0·7 µmol/L^(2)^. There is evidence from nationally representative surveys that approximately 80 % of pre-school children in India do not consume enough VA in their diet^(7)^. With an 18 % prevalence of low serum retinol status among pre-school children, VAD is considered a severe public health problem in India^(8)^.

In India, like many countries across the world, VA supplements are distributed to children under 5 years of age^(9)^, as this population group is particularly vulnerable to VAD. However, it is common for VAD to extend into the adolescent years^(2)^. Nutritional deficiency during childhood and adolescence is likely to be due to increased nutritional requirements for growth and development and represents a public health concern given the importance of VA during growth, skeletal development and sexual maturation^(1)^. The reported prevalence of VAD in adolescents in India is 15·6 %^(8)^

Vitamin A is found as retinol in animal-source foods such as meat and milk and occurs as provitamin A carotenoids in fruit and vegetables^(1)^. Provitamin A carotenoids can be cleaved and metabolised into retinol^(2)^. The Indian diet comprises primarily of pulses, coarse cereals and green leafy vegetables^(10)^ with low consumption of animal-source foods except milk and other dairy products^(11)^. Consequently, the main source of VA in the diet of Indian children and adults consists of milk, fruits and vegetables. However, it is known that the availability and affordability of fruits and vegetables follow seasonal patterns in many regions of the world^(12–15)^. A study involving women in Andhra Pradesh, south India, revealed that the intake of VA from food was almost 2-fold greater in the summer season (April) compared with during the rainy season (August)^(16)^. Low intake of VA-rich food can be linked to low serum retinol values and VAD. In a study involving lactating women in urban China, women whose fruit consumption was 200–400 g/day and > 400 g/day had higher serum retinol concentrations, respectively, compared with those who had a fruit consumption less than 200 g/day^(17)^ (*P* = 0·023 and *P* = 0·001, respectively). A study in northern Benin found that a lack of fruits and vegetables in the diet was significantly associated with VAD (adjusted OR = 2·72) in women^(18)^. As part of a study on the use of biofortified maize in Zambia, the study investigators reported that children living in areas where mangoes were readily available were suffering from hypercarotenaemia during mango peak season^(16)^. An analysis of their serum retinol concentrations confirmed that these children had higher body VA stores compared with children with no easy access to mango^(19)^.

The Comprehensive National Nutrition Survey (CNNS) was conducted in India from February 2016 to October 2018 and assessed the prevalence of micronutrient deficiencies, including VAD in pre-school children (1–4 years), school-aged children (5–9 years) and adolescents (10–19 years)^(8)^. Although the overall survey reported a 16 % prevalence of VAD among adolescents, certain areas showed rates exceeding 20 %, highlighting a significant public health problem^(8)^. The CNNS used cross-sectional methods and was therefore representative of specific geographic areas at the specific time periods of data collection^(20)^. As seasonality was not accounted for during data collection or later analysis, bias could have been introduced into the reported results^(20)^, especially if the nutritional status was linked to the consumption of foods that are likely to be highly seasonal. Seasonal variations have been seen to affect the nutritional status of adolescent girls in a study conducted in south India^(21)^. Understanding and factoring in seasonal fluctuations in food and nutrient intake is important when designing interventions to address micronutrient deficiencies in resource-limited settings^(22)^.

In this analysis, we aim to assess if the availability of VA-rich foods was associated with VAD, and if estimates of prevalence as reported in national reports might have been affected by the seasonal availability of VA-rich foods. Therefore, in this study, we considered the following research questions:

- Is there evidence of temporal variation in serum retinol in adolescents?
- Could this be driven by the seasonal availability of VA-rich food?
- Is there a difference by wealth quintile?
- What are the implications of the results for interpreting VAD prevalence based on CNNS data and the design of future surveillance?

## Materials and methods

### Data used

We undertook a secondary analysis of data from the CNNS, conducted by the Ministry of Health in India between 2016 and 2018. The survey assessed the nutritional status in pre-school (0–4 years), school-aged children (5–9 years) and adolescents. The CNNS employed a multi-stage design to select a representative sample of households and individuals from the three age groups across the thirty states in India. The survey included geographical stratification of urban and rural areas, followed by the selection of primary sampling units (PSU) using probability proportional to size sampling. A PSU comprised a minimum of 150 and a maximum of 300 households either within the same village or combined with the adjoining village. The survey is explained in greater detail in the CNNS report^(8)^.

Details on blood sampling and analysis are provided in the CNNS report. Briefly, blood samples collected for serum retinol analysis were protected from sun exposure and were analysed using HPLC reverse-phase chromatography at the designated laboratories. VAD was defined by serum retinol level < 0·7 µmol/L, marginal VA status by serum retinol between 0·70 and 1·05 µmol/L and replete VA status by serum retinol > 1·05 µmol/L. C-reactive protein (CRP) was measured using immunonephelometry, with two different kit-based methods of varying sensitivity, as described elsewhere^(23)^. The preliminary analysis of CRP data indicated a left-censored distribution (truncated at the limit of detection, 3·1 mg/L), arising from use of a low-sensitivity assay in the CNNS. The lack of reliable CRP data led us to exclude pre-school and school-aged children from the current analysis, as it is necessary to adjust for inflammation during assessment of their VA status^(24)^. However, adjusting for inflammation during assessment of VA status among adolescents is not recommended^(24)^, and we were able to proceed with biomarker data from 2297 adolescents. Although we did not adjust serum retinol concentrations for the effect of low-grade inflammation, higher levels of inflammation (as measured by CRP > 5 mg/L) were observed in 6 % of the adolescents, and sensitivity analyses were performed by excluding these individuals from the analysis.

To analyse the relationship between VA dietary intake and VA status, we intended to minimise the impact of factors that can confound this relationship, such as variations in disease prevalence or access to health care. We therefore selected states that were *a priori* comparable in terms of VA status but also where data were collected across more than one season. We first only retained states that were similar in terms of under-five mortality rate, as reported in the National Family and Health Survey 2019–2021 (online Supplementary Table 1), as this indicator is closely associated with VAD prevalence^(25)^. We then selected the category with the highest number of states, which corresponded to an under-five mortality rate of 25–35 deaths per thousand live births. Amongst these states, we only included states where data were collected across seasons. This led to the selection of the following states for the current analysis: Karnataka, Maharashtra, National Capital Territory of Delhi, Telangana and West Bengal. Sampling occurred in these states during February–September 2016–2018.

Sowing and harvesting periods are divided into two seasons: Kharif and Rabi^(26)^. Kharif crops are typically sown during the summer months of June and July and harvested in the autumn months of September and October, while Rabi crops are sown at the onset of winter in November and harvested during the spring months of March, April and May. The monsoon, which occurs during summer, or Kharif, is associated with changes in nutritional status^(20)^. The heavy rainfall associated with the monsoon period typically begins on the western coast at the end of May/beginning of June, advances across the entire country by mid-July and ends between September and early October^(27)^. The monsoon is critical for agriculture and rural livelihoods across South Asia and affects the availability of food items, including VA-rich fruits and vegetables. Therefore, we considered the months of June, July and August to represent the season of higher availability of VA-rich foods. We only selected states where some of the data collection occurred during this defined season (online Supplementary Table S1).

### Data analysis

To assess the availability of VA-rich foods by month and by state, we used dietary data from two population groups. The consumption data for meat, fish, milk, eggs, fruits and dark green leafy vegetables were available for the adolescent population group, in the form of the number of days per week they consumed a food group (values 0–7). However, some of the responses captured included ‘never’ and ‘occasional’, and we replaced these with numeric responses, that is, 0 and 2 d per week, respectively. Detailed data specifically on VA-rich fruit and vegetable consumption in adolescents were not available. Therefore, we used dietary data from children up to four years of age to calculate an indicator of VA-rich fruit and vegetable consumption at the PSU scale, by month. In the CNNS, these other VA-rich food items included fruits (ripe mango, jackfruit, cantaloupe) and roots and tubers (carrots, sweet potatoes, squash). Consumption of these food groups during the preceding day was measured in the age group 0–4 years through a food frequency questionnaire. Our assumption was that the consumption of VA-rich fruits and vegetables among children aged 0–4 years was a suitable proxy of consumption among the adolescent age group. We calculated the proportion of children who consumed these food groups. We assigned a value of ‘1’ to the children who consumed VA-rich fruits and foods and a value of ‘0’ to those who did not consume. Then we calculated the mean proportion of the children consuming VA-rich foods per PSU, and we considered this a potential explanatory variable.

We undertook an exploratory analysis of serum retinol concentration of adolescents with summary statistics, QQ plots and histograms. The data for serum retinol concentration were skewed, and it was necessary to transform them to their natural logarithms for further analysis. The summary statistics for retinol concentration, together with the QQ plots for the residuals from the exploratory model fitting with all the fixed effects, are shown in the Supplementary material (online Supplementary Table S2, Figs. S1, S2 and S3).

The linear mixed model framework was used to test different hypotheses about the relationship between serum retinol, seasonality and consumption of VA-rich foods. We considered the variables in Table 1 based on the strength of the plausible association between variables and serum retinol, derived from a technical consultation with a panel at the London School of Hygiene & Tropical Medicine, facilitated by a statistician. We added months to the model, followed by VA-rich foods in decreasing order of their VA content derived from the Indian food composition table^(28)^. Finally, we added wealth quintiles and age as we wanted to explore demographic and socioeconomic variation. *A priori* ranking was necessary to use the *α*-investment to control the false discovery rate and avoid problems associated with multiple hypothesis testing^(29)^.

**Table 1.** Sequence for predictors of serum retinol concentration for testing with *α*-investment

Order	Fixed effect	Unit/source of data
1	Month when child was sampled	Month/10–19 years
2	Meat and chicken	Frequency consumed within last 7 d/10–19 years
3	Vitamin A-rich vegetables/roots and tubers (e.g. carrots, pumpkin, sweet potatoes, squash)	Proportion of individuals who consumed in last 24 h/0–4 years
4	Vitamin A-rich fruits (e.g. ripe mangoes, papaya, jackfruit)	Proportion of individuals who consumed in last 24 h/0–4 years
5	Vitamin A-rich dark green leafy vegetables	Frequency consumed within last 7 d/10–19 years
6	Eggs	Frequency consumed within last 7 d/10–19 years
7	Fish	Frequency consumed within last 7 d/10–19 years
8	Milk	Frequency consumed within last 7 d/10–19 years
9	Other fruits	Frequency consumed within last 7 d/10–19 years
10	Wealth quintile of child	Wealth index/10–19 years
11	Age of child	Age in years/10–19 years

We sequentially tested the effect of the fixed effects (Table 1) within the linear mixed model framework, where the evidence that a fixed effect coefficient is significantly different from zero can be tested by using a Wald statistic^(30)^, which is obtained by computing a log-likelihood statistic:

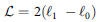




where 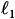 and 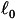 are the maximised log-likelihood from fitting the model with additional fixed effects and the simpler model without them, respectively. This statistic is an asymptotically distributed *χ*^2^ with *df* equal to the additional fixed effects. We started by fitting a ‘null’ model with serum retinol and the random effects, and this was fit by maximum likelihood. We refitted the model with the first fixed effect and computed the log-likelihood ratio statistic. We compared this model with the previous model, and if the *P*-value did not exceed 0·05, then the fixed effect was provisionally retained; otherwise it was dropped, and the next would be considered. When all the fixed effects were considered, we compared their *P*-values, and those with *P*-values that fell below threshold values under the false discovery rate control were retained. The final model was refitted by the residual maximum likelihood.

## Results

### Description of the population

All the 2297 adolescents with available biomarker data, CRP values and dietary consumption were included in the analysis from the five selected states. The mean age was 14 years. On average, VAD prevalence was approximately 10·3 % across all five states (Table 2). In subgroup analyses, CI were wide; however, VAD was higher in the younger age group (10–14 years) compared with the older age group (15–19 years), and VAD prevalence decreased with age. The prevalence of VAD was higher in the wealthier quintiles and in Telangana as compared with the other states.

**Table 2.** Descriptive statistics for the sampled population

	*n*	Vitamin A deficiency	
		Prevalence	CI 95 %
States			
Karnataka	324	8·3	4·9, 13·7
Maharashtra	315	11·2	7·0, 17·6
NCT of Delhi	597	11·8	6·9, 19·4
Telangana	386	19·5	14·9, 25·2
West Bengal	675	4·6	2·4, 8·4
Wealth quintiles			
Poorest	90	7·6	2·5, 20·7
Poor	237	6·2	3·6, 10·2
Middle	416	10·0	6·6, 14·9
Rich	664	13·7	10·2, 18·1
Richest	890	10·4	7·5, 14·2
Age group			
10–14	1221	12·7	9·9, 16·2
15–19	1076	8·1	6·1, 10·8
Sex			
Female	1092	11·3	8·6, 14·7
Male	1205	9·7	7·2, 12·8
Total	2297	10·3	8·3, 12·8

NCT, National Capital Territory.

### Seasonality of consumption of VA-rich food

**Fig. 1.**
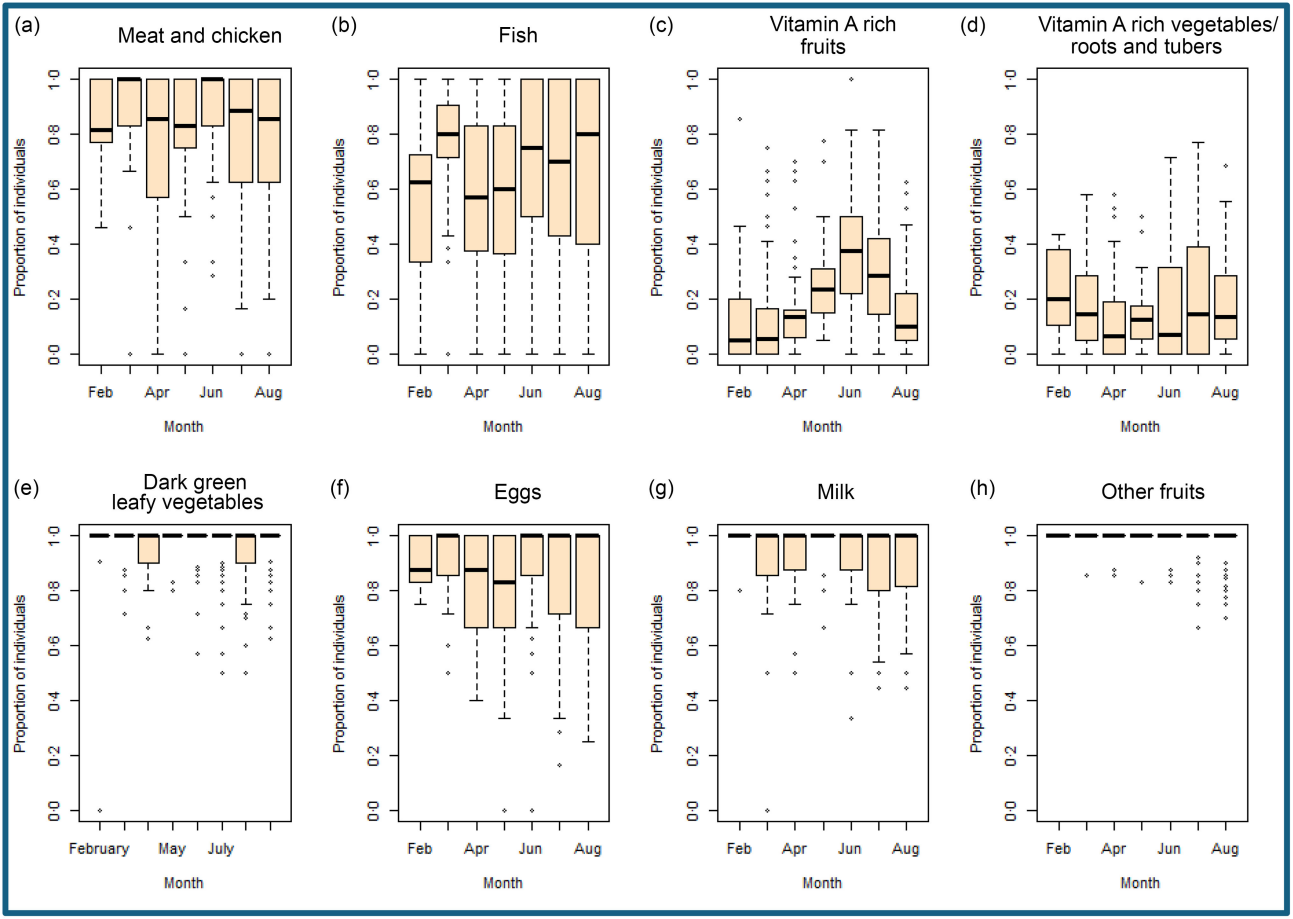
Boxplots showing the patterns and consumption proportion of the different food sources of vitamin A, across the five states.

Consumption of VA-rich foods was assessed among young children – aggregated by month at the PSU level – and was considered a proxy indicator of consumption among adolescents. The proportion of individuals who consumed different VA-rich food sources is shown in Fig. 1. The consumption of different VA-rich fruits (Fig. 1(c)) and VA-rich vegetables, roots and tubers (Fig. 1(a), (d)) varied seasonally, with peaks between May and September and in February. While < 10 % of children ate VA-rich fruits during most months, > 20 % of the children consumed VA-rich fruits during the summer and monsoon months (May–August), with a peak at 40 % in June. There were two peaks of consumption of VA-rich vegetables: one in February (20 %) and another one in June–August (about 18 %). For the other food sources (Fig. 1(a)–(b) and (e)–(h)), the consumption was relatively consistent throughout the year.

### Seasonality of vitamin A deficiency

**Fig. 2.**
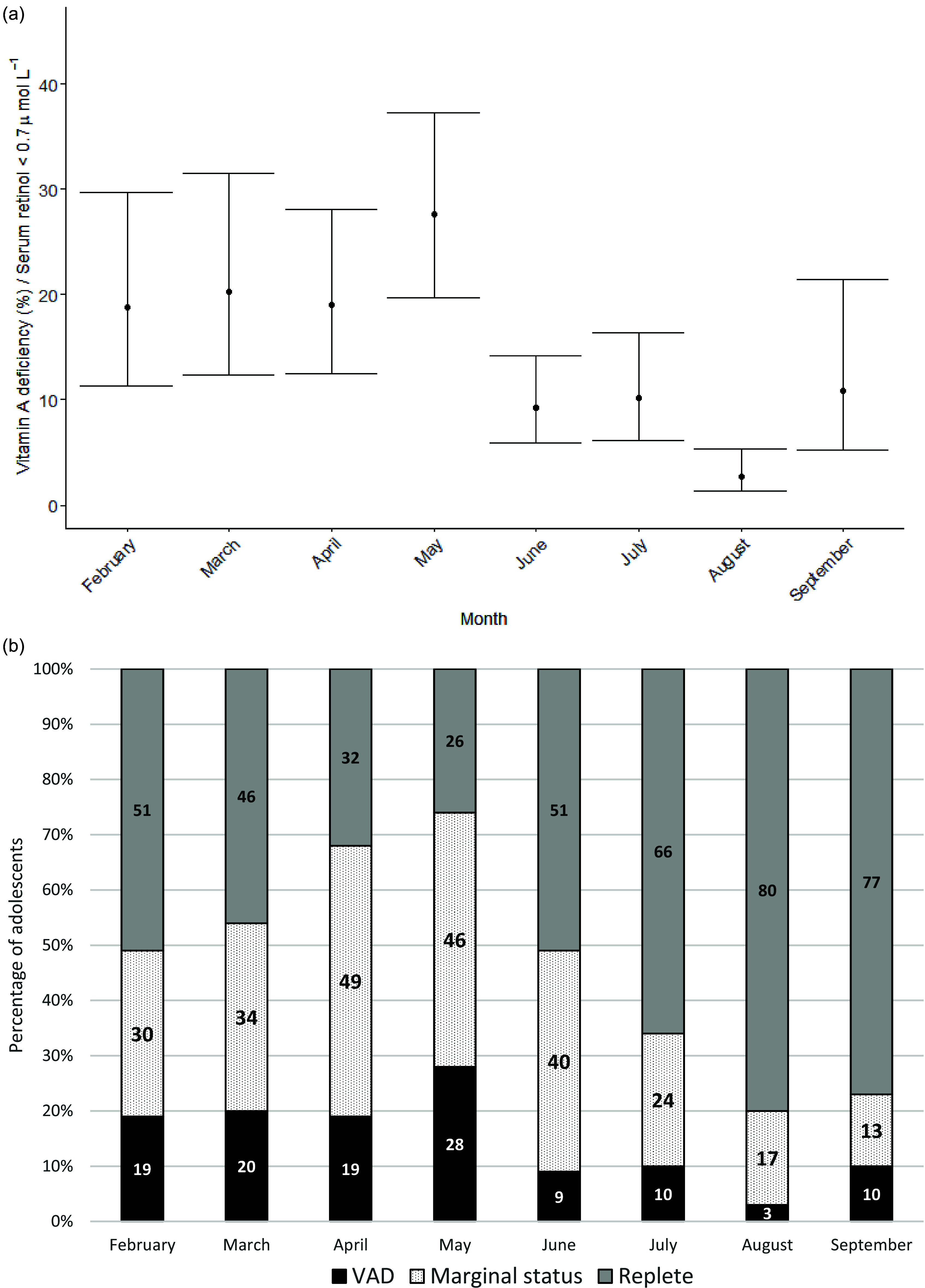
(a) Vitamin A deficiency (VAD) across five states. Points are average per month, and error bars are the 95 % CI. (b) Prevalence of VAD (in black), marginal VA status (black dots) and replete VA status (grey) by month in the five states. VAD is defined by serum retinol level < 0·7 µmol/L, marginal VA status by serum retinol between 0·70 and 1·05 µmol/L and replete VA status by serum retinol > 1·05 µmol/L.

The prevalence of VAD was greatest in May (28 %), decreased markedly from June and was at the lowest level in August (3 %) (Fig. 2(a)).

Marginal VA status, which indicates a level above deficiency but below repletion, was also the highest in April–May and the lowest in August–September. The prevalence of the population with a replete status of VA was greatest in August (Fig. 2(b)).

Excluding individuals with high inflammation, as measured by CRP > 5 mg/L, did not result in any substantive changes in deficiency prevalence estimates (less than 1·5 percentage point change for any given month, data not shown).

### Linear mixed model

**Fig. 3.**
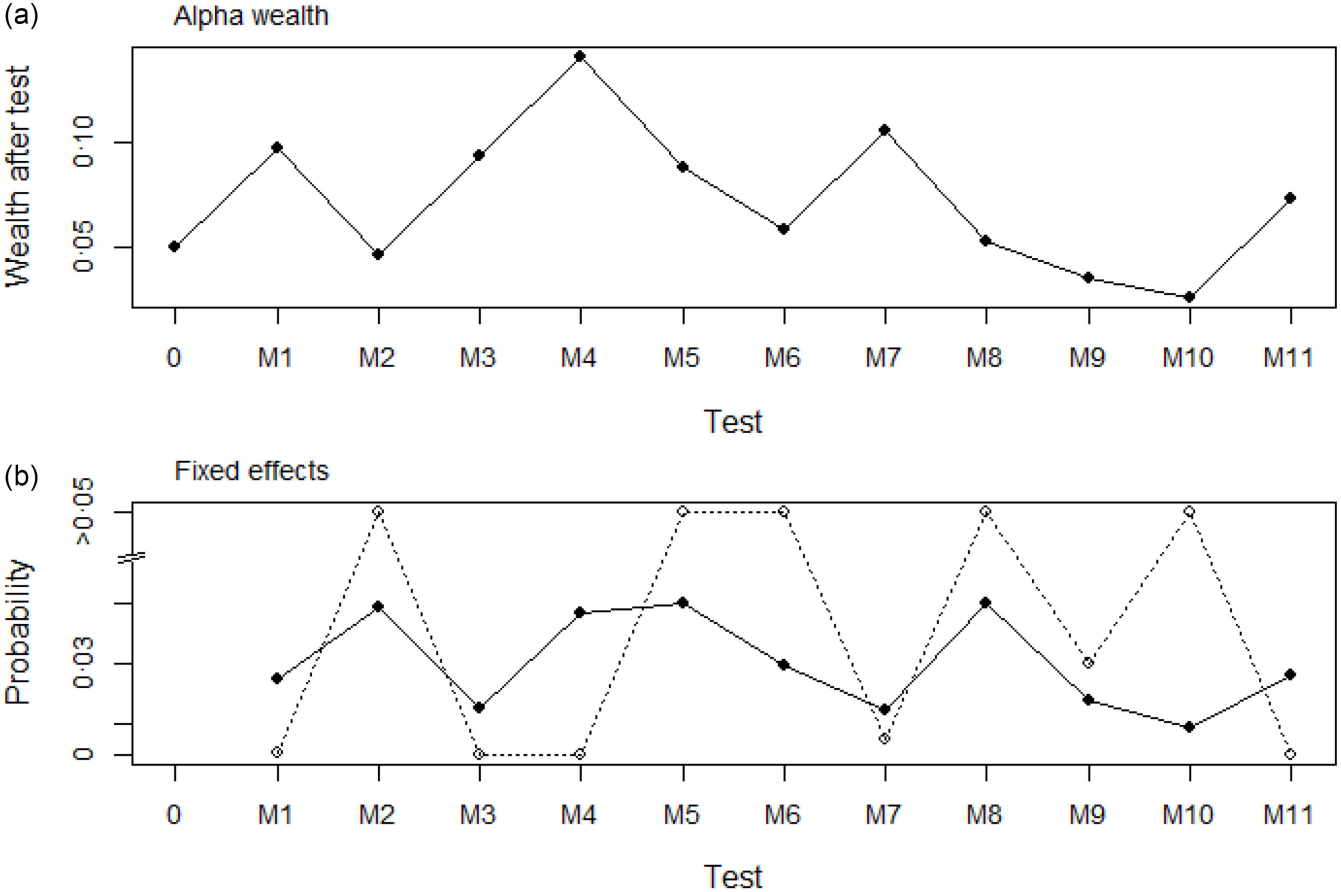
The ordered tests for fixed effect selection for serum retinol, the sequence of test is given in Table 1. (a) Filled circles shows the *α*-wealth after the successive tests, and (b) the open circles show the *P*-values of the successive tests and filled circles the corresponding thresholds values under the false discovery rate control.

Sequential fitting of the models, with maximum likelihood, resulted in the preliminary retention of the following fixed effects by ranking order: 1st (month when child was sampled), 3rd (VA-rich vegetables/roots and tubers), 4th (VA-rich fruits), 7th (fish), 9th (other fruits), 10th (wealth quintile) and 11th (age). However, when the *P*-values were compared with the thresholds according to *α*-wealth controlling the false discovery rate, only the following fixed effects were definitively retained in the model: month when child was sampled, VA-rich vegetables/roots and tubers, VA-rich fruits, fish and age (Fig. 3). The corresponding *P*-values for the order sequence of the test are shown in Fig. 3.

Serum retinol concentrations increased with age. The month of the year was also a significant factor, demonstrating a clear seasonal pattern, with the month of May being associated with the lowest concentration of serum retinol. Finally, greater consumption of VA-rich foods (fruits, vegetables/roots and tubers and fish) was associated with greater serum retinol. The coefficient estimates for the fixed effects of serum retinol (in log scale) with its standard error and *t*-value are presented in the Supplementary materials (see online Supplementary Table S3).

## Discussion

### Main findings

The VA status of adolescents in India was seasonally variable, appearing to be driven by the consumption of VA-rich fruits, vegetables, roots and tubers. In the five states selected for this analysis, the prevalence of VAD among adolescents was greatest in May (> 25 %) and lowest during June to August (< 10 %). There was a strong association between consumption of VA-rich foods and serum retinol in the linear model. High serum retinol levels coincide with the mango season in these states.

The design of the CNNS, in which different states were sampled in different months, raises the prospect of seasonal and spatial confounding. For the current analysis, we subset the CNNS sample to include states that were broadly comparable in < 5-year-old child mortality and where sampling occurred across seasons. Although this reduced our sample size, it increases our confidence that the associations between month of sampling and serum retinol status are driven by seasonal, rather than spatial, effects.

Furthermore, our results are consistent with patterns of production and consumption of VA-rich foods. In our selected states, almost 40 % of children reported consuming VA-rich foods the previous day (including mango, papaya, cantaloupe and jackfruit) during May–July, compared with < 10 % during the rest of the year. As mentioned above, the CNNS included detailed food consumption data for children (1–4 years) but not for adolescents, and food consumption data for children were considered a reasonable proxy for the adolescents. The variance in VA-rich food consumption can be explained by the availability and affordability of foods in relation to the rainy season, harvesting patterns and consumption preferences. As per the National Horticulture Board India, in the selected states, the period of harvesting mangoes usually begins by May (when the rainy season begins) and lasts until July–August^(31,32)^.

Our analysis indicates that retinol stores may contribute to adequate VA status during September–October, even as consumption of VA-rich foods declines. The liver has a large storage capacity for VA^(2)^, and high retinol intakes can build up stores that last for several months^(3)^ and be used during times of low dietary intake. It is, however, surprising that the level of deficiency increased significantly from August to September, rather than the prevalence of marginal status. However, the mean serum retinol value was equivalent in August and September, suggesting that many adolescents are close to the threshold values for deficient status. This could be further explored in longitudinal studies with repeat sampling on the same individuals.

The estimates of VAD were greatest for adolescents sampled in May, which is just before the beginning of the mango season. The drop in deficiency prevalence estimates between May and June is considerable yet biologically plausible. Total body VA stores regulate VA homeostasis, and low VA stores can upregulate the bioconversion of provitamin A carotenoids to retinol^(2)^, resulting in a rapid change in VA stores following high intake. It is consistent with previous published data showing sharp declines in VAD from 1 month to another in children in West Bengal^(33)^ and the significant increase in serum retinol concentrations observed in Indonesian children fed with fruit rich in carotenoids^(34)^. While there are no significant changes in deficiency prevalence between June and July, there is an increase in prevalence of VA repletion and a decrease in prevalence of VA marginal status, which suggests a sustained high intake of VA during these months, consistent with the dietary data that indicate a particularly high consumption of mangoes in June and July.

Serum retinol concentrations were elevated in about 10 % of adolescents (online Supplementary Fig. S1). This is unlikely to be due to a high consumption of VA-rich fruit and vegetables, as the body can downregulate the bioconversion of provitamin A carotenoids, and therefore, high intakes of VA-rich fruit and vegetables should not cause concern for hypervitaminosis^(2)^. These adolescents should not be receiving supplements according to national guidelines, and the exposure to fortified items was likely to be low at the time of the survey, given that the regulation for mandatory fortification of oil and milk was introduced in 2021. This is therefore a surprising finding; however, serum retinol is not considered an adequate indicator to define hypervitaminosis^(2)^, and we did not have any data on retinyl ester concentration to explore this further.

### Comparison with previous studies

The seasonal pattern of VA-rich fruit consumption is consistent with findings reported previously, including in low-income country contexts^(35)^, and seasonality in VAD has been reported previously^(27)^. In a study of 312 children (aged 0–4 years) in West Bengal, the authors reported two peaks of VAD: one in November–December and a larger one in May–June. There was a sharp decline in the prevalence of VAD starting in July and extending to October. The authors reported an increase in VA intake during July to September due to green leafy vegetable consumption, although mango consumption was not reported^(33)^. A study in Zambia reported that the consumption of mango was associated with a high level of serum retinol^(19)^. These studies are all consistent with the supposition that carotenoids present in the VA-rich foods are effectively converted into retinol, that serum retinol concentrations respond in a matter of weeks to increased consumption of VA-rich foods and that VA stores can maintain adequate serum retinol concentrations for approximately 2 months after VA-rich food consumption declines.

While some other studies globally have reported seasonal variation in the consumption of dark green leafy vegetables^(13)^, that was not evident in our analysis. We did not find any association between serum retinol levels and the consumption of dark green leafy vegetables, milk or eggs, which is contrary to a study in south India where dark leafy vegetables were the major source of VA during the rainy season^(16)^. There was, however, a positive association between the concentration of serum retinol in adolescents and consumption of fish. We also observed a positive association between serum retinol in adolescents and the consumption of VA-rich roots and tubers in children less than 5 years of age, which served as a proxy of the consumption pattern of adolescents. Carrots, a rich source of VA, are available mostly during the winter months^(36,37)^, and that was reflected in our results with an increase in consumption during February (Fig. 1(d)). Along with this, our results showed another peak of VA-rich food consumption in July with an increased proportion of children consuming VA-rich roots and tubers, which can be attributed to the fact that sweet potatoes are available in monsoon and in winters^(38–40)^. Our study findings resonate with previous studies conducted globally and in the Indian subcontinent that establish seasonality as an important driver of dietary micronutrient intakes^(41–44)^.

Seasonality is an important aspect to consider in population VA assessment and has been recognised as a key driver of dietary diversity, food availability and household food security in many low- and middle-income countries^(41)^. Studies have indicated a positive correlation of plasma retinol with normal monthly rainfall and negative correlations with seasonal changes in mean temperature and solar UV radiation, suggesting that environmental conditions may also affect circulating levels of retinol^(45)^. Therefore, it is highly probable that VAD, as reflected by serum retinol levels, is profoundly influenced by seasonal variations.

### Strengths and limitations

A strength of the analysis is the large sample size (despite the need to draw a subset of the CNNS sample), the availability of individual-level micronutrient biomarker data and the triangulation of data sources to investigate food system drivers of VA status. The analysis avoided potential confounding due to high-dose VA supplementation, since only young children are included in this programme in India. Furthermore, exposure to milk and oil fortification with vitamin A was not likely to be high at the time of CNNS data collection. Future studies may include other age groups to broaden the policy relevance, and it will be important to ensure robust measurement of CRP in future surveys.

A limitation of the analysis is the cross-sectional nature of the data and the lack of detailed consumption data for adolescents. We therefore relied on analysing consumption data for children and treating this as a proxy variable for food consumption among adolescents. The findings on consumption of VA-rich foods, including seasonality of fruits and vegetables, were consistent with seasonal patterns of production, and we do not expect our main findings would be substantively different if individual-level food consumption data were available for adolescents.

We were not able to assess the impact of low-grade inflammation on serum retinol concentrations, due to the lack of sensitivity of the CRP assay at low values of CRP (under 3·1 mg/L). However, the small number of adolescents with high value of CRP (above 5 mg/L) indicates a low prevalence of inflammation in this population.

### Policy relevance

The focus on adolescents is highly relevant for public health policy, given the importance of VA status for healthy growth and development. Unlike young children, adolescents do not receive high-dose VA supplementation in India, and adequate VA status therefore relies on dietary sources, which likely means that VA status is more seasonally variable and potentially of greater public health relevance among adolescents than children. Our findings also indicate a higher prevalence of VAD among adolescents from wealthier groups. This observation may be attributed to dietary patterns among wealthier adolescents, who tend to consume more processed, nutrient-poor foods, leading to a reduced intake of vitamin A-rich foods^(46–48)^. We also noticed that the prevalence of VAD was higher in younger adolescents (10–14 years) compared with the older age group (15–19 years), which is in line with evidence showing higher needs for vitamin A during growth^(1)^.

Our findings support a nuanced interpretation of survey findings from the CNNS, as seasonality of VA-rich food consumption was not accounted for in the survey results. Two out of the three states that were identified as deficient to a level of public health concern in the CNNS main report were sampled outside the VA-rich food season, and our findings suggest that VAD prevalence would have been lower had the sampling been conducted during the VA-rich food season. Similarly, most of the states considered as VA replete were sampled during the VA-rich food season. This suggests that limited, but statistically grounded, sampling during alternative seasons – in seasons with low and high availability of VA-rich foods – could help establish the extent of any inter-seasonal variation. Knowledge of seasonal variations in VAD is important to understand the determinants of VA status and to design effective interventions to prevent and control VAD. As subnational policies can be put in place in response to the CNNS findings, it is important to consider factors that can confound the assessment of VAD. Our results demonstrate that seasonality should be considered in the design and analysis of biomarker and dietary surveys to assess VA status and in the monitoring and evaluation of programmes and interventions to reduce VAD.

The Global Alliance for Vitamin A recommended that scaling back VA supplementation could occur in vulnerable populations when the government can verify a low prevalence of VAD and sustainable intake of VA throughout the year^(49)^. The scaling back of VA supplementation has been subject to extensive debate in India over recent years^(5,50)^. Through the triangulation of biomarker and food system data, the findings of the current analysis support continued VA supplementation in India, although seasonal deployment is likely to be more effective. The analysis should be replicated in younger age groups to provide greater certainty in support of decisions on VA supplementation. The study could also be repeated using data from the Diet and Biomarker Survey of India, when these become available, which will capture the changing context of diets and fortification of food items.

Although it is recommended that surveys evaluating the prevalence of wasting in a country are conducted in the same month each year^(51)^, there are other factors like timing of the surveys, including regional and cultural differences while designing the surveys and statistical adjustments that can be used to account for seasonality^(52)^ in the implementation of national nutrition surveys or in the interpretation of survey results. This is especially relevant for a large country like India, which has significant seasonal variation across different regions and hence variance in availability of VA-rich foods. Incorporating seasonality into survey design could result in a more comprehensive understanding of dietary habits and nutrient intake, leading to more effective public health interventions and policies aimed at improving nutrition outcomes.

### Conclusion

Our study demonstrates large seasonal variation in the VA status of adolescents in India, which can be explained by the availability of VA-rich foods. The prevalence of VAD among adolescents was ≥ 19 % during February–May but ≤ 10 % during June–September, which coincides with the monsoon season and greater availability of VA-rich foods. The prevalence of marginal VA status followed the same seasonal trends. The findings demonstrate that VAD remains a public health issue in India, indicating that current food systems and public health nutrition strategies including large-scale food fortification were not meeting dietary VA requirements among adolescents at the time of the CNNS. This is concerning given the importance of VA for growth, skeletal development and sexual maturation. If similar findings are observed in younger children, this would suggest that the VA supplementation programme could be seasonally deployed to increase effectiveness. The contribution of large-scale food fortification to dietary VA intakes is likely to have increased since the time of the CNNS, and it would be worth re-assessing the seasonality of VA status when data become available from the recent national Diet and Biomarker Survey of India.

Our study demonstrates the importance of incorporating seasonality considerations into nutrition survey design in India. Failure to account for seasonality in survey design could lead to inaccurate estimation of population VA status and VAD prevalence, including at national and subnational scales. For example, in the CNNS, apparent differences in VAD prevalence between states may be an artefact of survey design, as states were sampled at different times of the year and need careful interpretation. Similarly, seasonality should be incorporated into the monitoring and evaluation of interventions and programmes to improve VA status (including supplementation and fortification). Finally, our study demonstrates the value of integrating nutrition and food systems perspectives to improve our understanding of population micronutrient dynamics and the drivers of micronutrient deficiency risks.

## Supporting information

Sahota et al. supplementary material Sahota et al. supplementary material

## Supplementary material

For supplementary material/s referred to in this article, please visit https://doi.org/10.1017/S0007114525103681.
