# Supplementary material for: Vitamin A deficiency in India and seasonality of vitamin A-rich food consumption
Source: Br J Nutr. 2025 Jun 23;133(12):1522–31. doi: 10.1017/S0007114525103681 (PMC12335917; doi:10.1017/S0007114525103681)
Supplement: Sahota et al. supplementary material [file S0007114525103681sup001.docx]

**Supplementary Information**

Table S1: Under-5 mortality rate and sample collection time in the 29 states of India

| **States** | **U5MR** | **U5MR category** | **Sample collection time for the  states in the 25-35 category** | **selection** |
| --- | --- | --- | --- | --- |
| Andhra Pradesh | 35 | 35-45 |  |  |
| Arunachal Pradesh | 19 | <20 |  |  |
| Assam | 39 | 35-45 |  |  |
| Bihar | 56 | >50 |  |  |
| Chhattisgarh | 50 | >50 |  |  |
| Delhi | 31 | 25-35 | March-September | x |
| Goa | 11 | <20 |  |  |
| Gujarat | 38 | 35-45 |  |  |
| Haryana | 39 | 35-45 |  |  |
| Himachal Pradesh | 29 | 25-35 | July-October |  |
| Jammu and Kashmir | 19 | <20 |  |  |
| Jharkhand | 45 | 40-50 |  |  |
| Karnataka | 30 | 25-35 | June-September | x |
| Kerala | 5 | <20 |  |  |
| Madhya Pradesh | 49 | 40-50 |  |  |
| Maharashtra | 28 | 25-35 | November-May | x |
| Manipur | 30 | 25-35 | January-April |  |
| Meghalaya | 40 | 35-45 |  |  |
| Mizoram | 24 | 15-25 |  |  |
| Nagaland | 33 | 25-35 | January-May |  |
| Odisha | 41 | 40-50 |  |  |
| Punjab | 33 | 25-35 | September-February |  |
| Rajasthan | 38 | 35-45 |  |  |
| Sikkim | 11 | <20 |  |  |
| Tamil Nadu | 22 | 15-25 |  |  |
| Telengana | 29 | 25-35 | March-July | x |
| Tripura | 43 | 40-50 |  |  |
| Uttar Pradesh | 60 | >50 |  |  |
| Uttarakhand | 46 | 40-50 |  |  |
| West Bengal | 25 | 25-35 | June-September | x |
| U5MR: Under 5 mortality rate (number of deaths per 1,000 live births)  source: NFHS 5 (2019-2021) as reported in Economic survey 2022-2023 | | | |  |

Table S2 Summary statistics for retinol concentration, together with residuals from the exploratory model fitting with all fixed effects.

|  | Serum retinol  (µmol/L) | Residuals from model, serum in retinol in original units | Residuals from model, log transformed retinol |
| --- | --- | --- | --- |
| Mean | 1.32 | -0.00 | 0.00 |
| Median | 1.19 | -0.10 | -0.01 |
| Standard deviation | 0.63 | 0.57 | 0.41 |
| Skewness | 1.34 | 1.17 | -0.02 |
| Octile skewness | 0.25 | 0.21 | -0.00 |

Table S3 Coefficient estimates for the fixed effects of serum retinol (in log scale) with its standard error and *t*-value.

| Fixed Effect | Coefficient estimate | Standard error | t value |
| --- | --- | --- | --- |
| Intercept | 0.041 | 0.152 | 0.272 |
| Month (February) | -0.289 | 0.142 | -2.040 |
| Month (March) | -0.239 | 0.129 | -1.847 |
| Month (April) | -0.217 | 0.133 | -1.623 |
| Month (May) | -0.338 | 0.142 | -2.384 |
| Month (June) | -0.117 | 0.143 | -0.819 |
| Month (July) | -0.136 | 0.143 | -0.956 |
| Month (August) | -0.059 | 0.141 | -0.422 |
| Month (September) | -0.024 | 0.142 | -0.170 |
| Month (October) | -0.254 | 0.216 | -1.174 |
| Month (November) | -0.176 | 0.152 | -1.159 |
| Month (December) | -0.018 | 0.150 | -0.118 |
| Vitamin A rich tubers and squashes | 0.076 | 0.107 | 0.709 |
| Vitamin A rich fruits | -0.001 | 0.094 | -0.011 |
| Fish | 0.018 | 0.006 | 2.794 |
| Age | 0.016 | 0.003 | 5.744 |


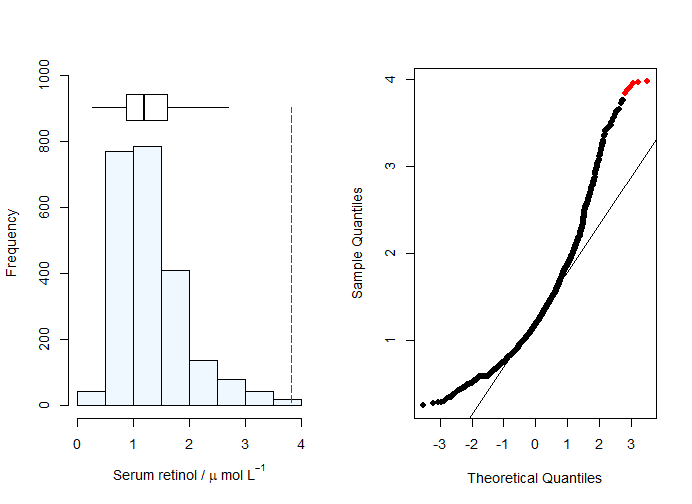


Figure S1: Histogram and QQ plots for serum retinol concentration in adolescents.


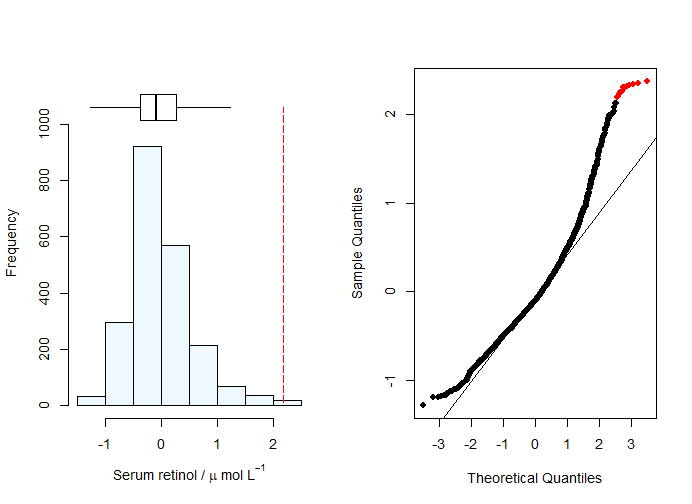


Figure S2 Histogram and QQ plot for the residuals from an exploratory fit of the saturated model (all potential fixed effects) for the concentration of serum retinol in adolescents.


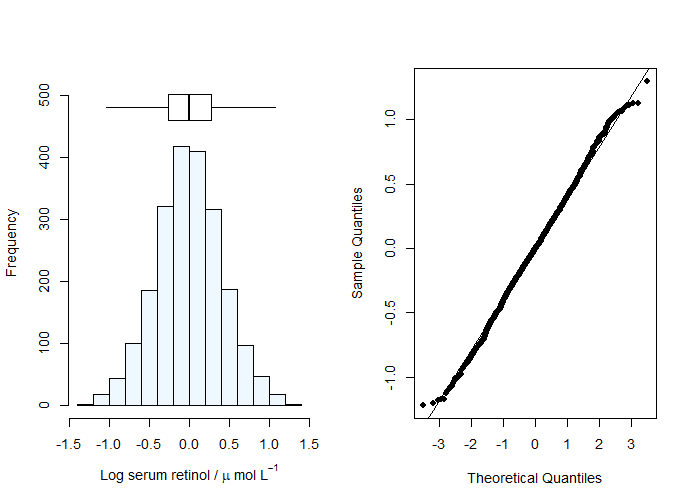


Figure S3 Histogram and QQ plot for the residuals from an exploratory fit of the saturated model (all potential fixed effects) for the concentration of serum retinol in adolescents.


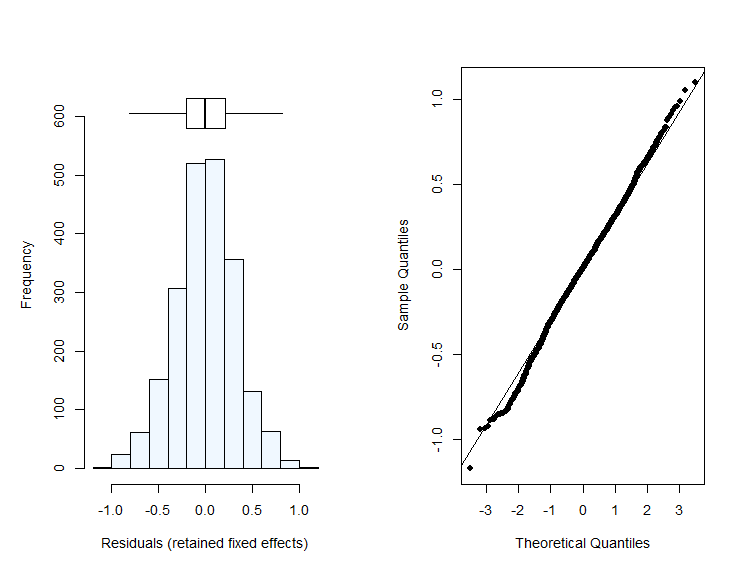


Figure S4: Histogram and QQ plot for the residuals from an exploratory fit of the final model (retained fixed effects) for the concentration of serum retinol in adolescents.


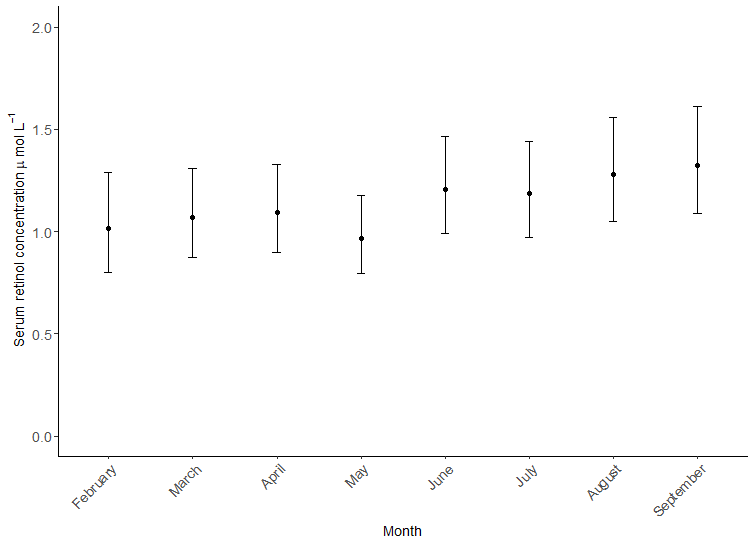


Figure S5*:* Serum retinol concentration by month (mean, 95% CI) across the five states for the adolescent group.
